# Keep the quality high: the benefits of lot testing for the quality control of malaria rapid diagnostic tests

**DOI:** 10.1186/s12936-020-03324-3

**Published:** 2020-07-13

**Authors:** Sandra Incardona, David Bell, Ana Campillo, Jane Cunningham, Frederic Ariey, Thierry Fandeur, Jennifer Luchavez, Christian Anthony Luna, Didier Ménard, Sina Nhem, Johanna Beulah Sornillo, Benoit Witkowski, Zachary Katz, Sabine Dittrich, Xavier C. Ding

**Affiliations:** 1 FIND, Geneva, Switzerland; 2 Independent consultant, Issaquah, WA USA; 3 World Health Organization/Global Malaria Programme (WHO/GMP), Geneva, Switzerland; 4 Hôpital Cochin, Laboratoire de Parasitologie-Mycologie, INSERM U1016 (Institut Cochin), Université de Paris, Paris, France; 5 Direction Internationale, Institut Pasteur de Paris, Paris, France; 6 Parasitology Department, Research Institute for Tropical Medicine, Muntinlupa, Philippines; 7 Malaria Genetics and Resistance Unit, Parasites and Insect Vectors Department, Institut Pasteur, Paris, France; 8 National Center for Entomology, Parasitology and Malaria Control, Phnom Penh, Cambodia; 9 Epidemiology and Biostatistics Department, Research Institute for Tropical Medicine, Muntinlupa, Philippines; 10 Laboratoire d’Epidémiologie Moléculaire du Paludisme, Institut Pasteur du Cambodge, Phnom Penh, Cambodia; 11 Nuffield School of Medicine, University of Oxford, Oxford, UK

**Keywords:** Malaria, Diagnostics, Rapid diagnostic test, Lot testing, Quality control, Post-market surveillance

## Abstract

**Background:**

The production and use of malaria rapid diagnostic tests (RDTs) has risen dramatically over the past 20 years. In view of weak or non-existing in vitro diagnostics (IVD) regulations and post-marketing surveillance (PMS) systems in malaria endemic countries, the World Health Organization, later joined by the Foundation for Innovative New Diagnostics, established an independent, centralized performance evaluation and Lot Testing (LT) programme to safeguard against poor quality of RDTs being distributed through the public health sector of malaria endemic countries. RDT performances and manufacturer quality management systems have evolved over the past decade raising questions about the future need for a centralized LT programme.

**Results:**

Between 2007 and 2017, 6056 lots have been evaluated, representing approximately 1.6 Billion RDTs. A total of 69 lots (1.1%) failed the quality control. Of these failures, 26 were detected at receipt of the RDT lot in the LT laboratory, representing an estimated 7.9 million poor quality RDTs, and LT requesters were advised that RDTs were not of sufficient quality for use in patient management. Forty-three were detected after long-term storage in the laboratory, of which 24 (56%) were found to be due to a major issue with insufficient buffer volume in single use buffer vials, others predominantly showing loss of sensitivity. The annual cost of running the programme, based on expenses recorded in years 2014–2016, an estimated volume of 700 lots per year and including replenishment of quality control samples, was estimated at US$ 178,500 ($US 255 per lot tested).

**Conclusions:**

Despite the clear benefits of the centralized LT programme and its low cost compared with the potential costs of each country establishing its own PMS system for RDTs, funding concerns have made its future beyond 2020 uncertain. In order to manage the risks of misdiagnosis due to low quality RDTs, and to ensure the continued safety and reliability of malaria case management, there is a need to ensure that an effective and implementable approach to RDT quality control continues to be available to programmes in endemic countries.

## Background

**Fig. 1:**
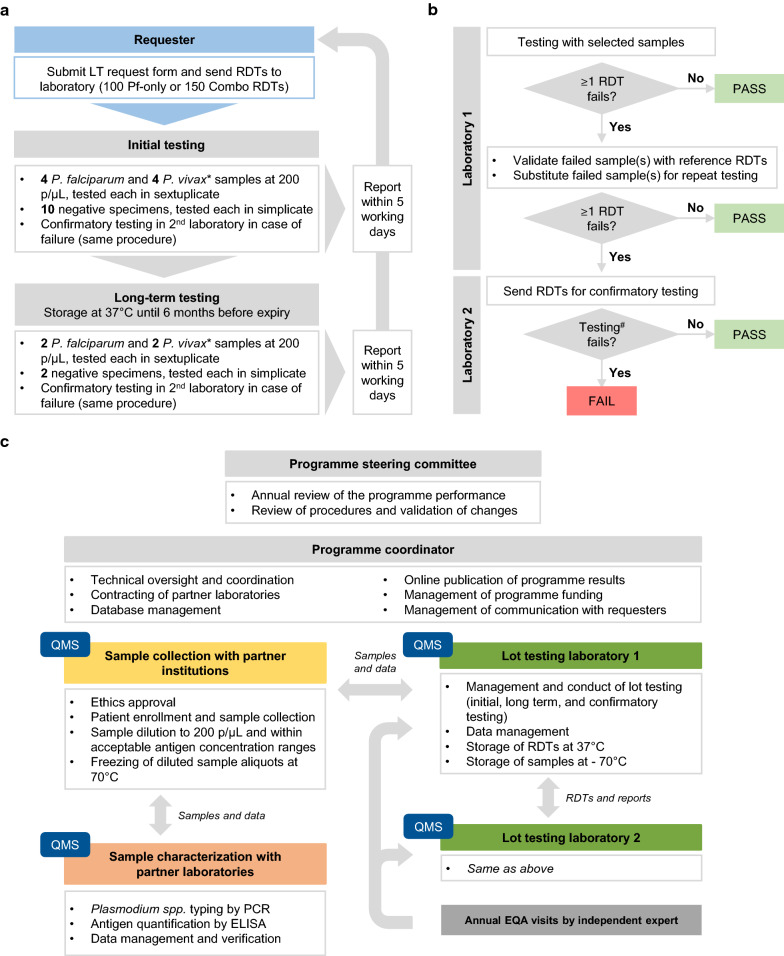
Overview of the Lot Testing process. **a** Schematic overview of the overall lot testing process, based on samples at 200 parasites per microlitre of blood (p/µL) and with antigen concentrations within a standardized range corresponding to this parasitaemia, *indicates a condition that applies to combination tests only. **b** Schematic overview of the testing procedure and pass/fail criteria. RDTs must detect all repeats of all samples at 200 p/µL in order to pass. False positives and anomalies, such as red background, flow failure, etc., are reported as comments and not used for pass/fail decisions. Insufficient buffer is reported as a “fail” result, ^#^confirmatory testing in a second laboratory, if necessary, is performed according to the same two-step procedure than for the initial testing and using a different set of samples. **c** Main components and activities required for the LT programme are indicated. LT = Lot Testing, Pf = *P. falciparum*, EQA = External quality assessment, QMS = Quality management system

As RDTs became increasingly used in the early 2000s, critical quality limitations became apparent [1–3] which could not be identified and resolved by the weak regulatory oversight in endemic countries. To respond to this need for an independent quality control (QC) system, the World Health Organization (WHO), together with the Research Institute for Tropical Medicine, Philippines, and the Institut Pasteur du Cambodge, Cambodia, initiated a lot-testing (LT) programme in 2003, which then expanded in collaboration with the Foundation for Innovative New Diagnostics (FIND) to a Global RDT Evaluation Programme, comprising the Product Testing and LT programmes [4, 5]. The former informed WHO procurement criteria until RDT prequalification became a requirement in December 2017. LT assesses lot quality before deployment at the request of procurers, manufacturers, or National Malaria Control Programmes (NMCPs), using a testing algorithm with standardized QC samples derived from patient blood [6] (Fig. 1).

Within a few years, LT has become a component of the procurement processes of all major public sector procurers, including Médecins Sans Frontières (MSF), the United States President’s Malaria Initiative (PMI), and the United Nations International Children’s Emergency Fund (UNICEF), and it became mandatory for procurement under grants from the Global Fund (GF). When the funding of the UNITAID programme ended in 2017, the WHO leveraged funding to support one laboratory, but stopped replenishing QC sample stocks and the future of the programme is now unclear [7]. Since mid-2017, the GF ceased to require WHO-coordinated LT, requesting instead that countries “arrange for the monitoring of the quality of diagnostic products procured with grant funds in line with relevant WHO guidelines on post-marketing surveillance (PMS) of in vitro diagnostics (IVDs)” [8]. Precise guidance on testing procedures and QC standards is however not available, except some example procedures for LT of HIV RDTs [9]. Other procurers continue to utilize the service.

### Impact of the lot-testing programme

From 2007 to 2017, 6056 lots were evaluated, representing an estimated 1.6 billion RDTs [10] (unpublished LT programme data). Overall, 69 (1.1%) failures were detected, of which 26 at RDT receipt and 43 after long-term storage (Table 1). The 26 initial failures correspond to an estimated 7.9 million RDTs, which could have reached end-users and potentially led to incorrect clinical decisions. Most failures (20/26, 76.9%) were due to non-detection of *Plasmodium vivax* samples at 200 parasites/µL, and failure rates did not change much over time, demonstrating the importance of continued testing.

**Table 1:** Summary of Lot Testing failures (2007–2017)

Reasons for lot failure	Nb lots failed at reception (nb RDTs)	Nb lots failed after long-term storage (nb RDTs)
Non-detection of Pv at 200 p/μl	20 (5.7 M)	18 (5.1 M)
Non-detection of Pf and Pv at 200 p/μl	0	1 (0.3 M)
Insufficient buffer^a^	6 (1.7 M)	24 (6.8 M)
Total	26 (7.4 M)	43 (12.2 M)

The numbers of lot failures and –in brackets-the corresponding estimate of numbers of RDTs (based on a mean lot size of 283,053 RDTs per lot, as communicated by LT requesters from 2013 to 2017) are shown for different reasons of failed testing (i.e. confirmed negative test results with *P. falciparum* (Pf) and *P. vivax* (Pv) samples standardized at a density of 200 parasites per microlitre of blood (p/µl), which were either observed upon reception of the RDT lot or after long-term storage

*Nb* number, *M* million

^a^Counted as a failure only from June 2016 onwards

More than half of the long-term failures (24/43, 56%) were due to partly evaporated buffer in single-use buffer vials [10–12]. Throughout 2014 to June 2017, this problem affected 24 lots of 5 products from 3 WHO-prequalified manufacturers. The detection of this issue triggered a WHO Notice of Concern discouraging procurement of these products until problem resolution [13]. Without LT, such issues may go undetected, as proactive post-marketing surveillance (PMS) for diagnostics is weak and health workers are usually not trained to detect and report quality issues, often being unaware of existing complaint systems [14, 15]. The programme’s formal reporting of RDT anomalies, such as incomplete clearing and red background [16], also formed the basis for successful product replacement in some cases. The ‘non-routine’ LT assessed RDTs withdrawn from the field in various countries and allowed differentiating between product defects, operator errors, and parasite factors. Ruling out product defects led to the subsequent confirmation of high *Plasmodium falciparum hrp2/hrp3* gene deletion rates in Eritrea as the cause of false negative RDT results [17], impacting on the use of HRP2-based RDTs in this region.

There is a high probability that the LT programme has also impacted on RDT quality through the pressure placed on manufacturers to meet LT quality standards for all lots sold in the public sector, and/or through the potential publicity associated with failure. A lot failure typically leads to lot replacement and is associated with financial and reputational costs for manufacturers. In a survey conducted in 2014, 31% (11/35) of manufacturers indicated that the LT Programme had triggered improvements of their lot-release procedures or QC panel characteristics [18].

### The future of LT

These findings demonstrate important contributions of the LT programme detecting defects even in WHO prequalified products. Other existing processes verifying RDT quality cannot fully substitute for such an independent programme. While manufacturers have their own lot-release procedures, assessed by the WHO prequalification of diagnostics (PQ) process [19], there is no established mechanism to ensure continued adherence to good manufacturing practice and PMS after a product is prequalified. Furthermore, it is the responsibility of the NMCPs and National Regulatory Authorities (NRAs) to perform PMS, but the processes are poorly defined or essentially non-existent in most low resource countries [14, 15].

India and Nigeria have established their own LT systems through collaborations with the WHO/GMP and FIND, respectively, using samples prepared locally as per the publicly available WHO-FIND procedures [20]. Other countries may adopt such a system, however the running costs, the complexity of preparing and characterizing QC samples and ensuring laboratory certification represent a considerable investment in light of the infrequent testing need of a single country. QC samples for RDTs need to have known antigen concentrations but validated antigen quantification methods are not routinely available in endemic countries. From the manufacturer’s or RDT procurer’s point of view, it may be challenging to deal with different laboratories and turnover times for individual countries. This does not obviate the importance of more country ownership and building efficient capacity at country or regional level to manage product quality—including post-distribution quality monitoring—across not just malaria diagnostics but those for other diseases. However, this capacity has to be matched against overall programme costs, and the ability to verify quality of the testing process and site.

The WHO-FIND system used instead an international network of reference laboratories with centralized sample characterization (Fig. 1) and is the only example of a centralized, internationally-recognized LT programme for diagnostics. The main challenges faced, e.g. developing and confirming samples of known reactivity, adapting sample collection sites and periods to changing malaria epidemiology, or facing low sample outputs within the required antigen concentration ranges, were easier to absorb with the centralized mechanism than it would have been for a country-specific programme with limited funding.

Financially, the centralized LT programme is probably the most cost-effective investment. Based on expenses incurred for the 2014–2016 period, the mean cost per lot tested has been estimated to US$ 255 (Table 2). With approximately 700 lots tested per year, this translates to an annual cost of around $US 178,500 in a market of over 400 million tests and an estimated budget of 120 million USD annually [21, 22]. This seems a small price to pay to prevent incorrect diagnoses with poor quality RDTs, and to maintain confidence in RDT results.

**Table 2:** Estimation of costs for Lot Testing (2014–2016)

Cost estimate (US$)	Description
375,487.08	LT functioning^1^
31,341.43	QC sample collections^2^
39,043.74	Characterization of samples^2^
197,425.20	Personnel
19,117.33	Operational costs
662,414.79	Total costs 2014–2016
254.48	Costs per lot^3^

Costs for LT were estimated based on expenses incurred by the Global RDT Evaluation Programme from 2014 to 2016, by extracting payments specifically done for LT-related activities

^1^ Budgets for LT work in the WHO-FIND reference laboratories, procurement of materials for LT, etc

^2^ Prorata of overall Programme’s costs for QC samples based on percentage of samples used for LT

^3^ Based on 2603 lots tested throughout 2014–2016

### Building a framework for RDT quality control

The LT programme is only one part of an intervention framework that ensures RDT quality. Ideally, the NRAs and NMCPs should implement and support proactive and reactive PMS activities with adequate capacity and funding, for which major donors can play a crucial role.

A number of useful resources are publicly available (Table 3), such as the WHO guidelines for PMS of IVDs [9], a troubleshooting guide for frequently observed anomalies and errors [23], and a protocol to help setting up a process for detecting, investigating, and acting upon quality issues [24]. Training manuals, videos, job aids and quizzes have been developed by the WHO, some with extensive field testing in Zambia [25–27]. Reference materials also exist, including panels of culture-derived *P. falciparum* parasites [28], international standards [29], or HRP2, pLDH and aldolase recombinant proteins [30, 31], to be used as such or to develop secondary reference materials. These well characterized, easy to handle and temperature-stable materials may be used in complement to LT with patient-derived samples, e.g. for cross-checking RDTs withdrawn from the field, however standard procedures for their use need to be developed and widely accepted. Panels of pre-diluted recombinant proteins have been evaluated with national reference laboratories and found easy to use for RDT QC (FIND, unpublished work).

**Table 3:** List of available resource to support the PMS and correct use of malaria RDTs

Type of resource	Resource	Access
Guidance	Post-market surveillance of in vitro diagnostics	https://apps.who.int/iris/bitstream/handle/10665/255576/9789241509213-eng.pdf;jsessionid = E93138DC88FDD4ABD2D215B6406002AB?sequence = 1
SOP	Methods manual for laboratory quality control testing of malaria RDTs	https://www.who.int/malaria/publications/rdt-lab-quality-manual/en/
SOP	Protocol on responding to problems with malaria RDTs	https://www.finddx.org/wp-content/uploads/2016/10/Malaria-RDT-protocol-24JUN16-FINAL.pdf
Guide	Troubleshooting guide for supervisors overseeing users of malaria RDTs	https://www.finddx.org/wp-content/uploads/2016/10/RDT-supervisors-guide-2016.pdf
Form	User complaint form for reporting problems and/or adverse events related to diagnostic products	http://www.who.int/diagnostics_laboratory/procurement/111121_user_complaint_form_for_adverse_events_and_product_problems_reporting_english.pdf?ua=1
Ref material	*Plasmodium falciparum* antigens (1st International Standard)	https://www.nibsc.org/products/brm_product_catalogue/detail_page.aspx?catid=16/376
Ref material	*Plasmodium falciparum* culture-derived panels	https://www.zeptometrix.com/categories/assay-developers/microorganisms filtered by “malaria”
Ref material	*Plasmodium falciparum* and *P. vivax* recombinant antigens (HRP2, pLDH, aldolase)	https://www.microcoat.de/Products/malaria-reference-materials-p-falciparum-hrp-2-recombinant/ https://spandiag.com/recombinant-antigens-for-diagnostics/
Training material	Training manuals, job aids, results guides, quizzes and answer keys: available as generic versions for *P. falciparum*-only, Pf-pan and pan-Pf RDTs, and as RDT product-specific versions.	https://www.finddx.org/reports-and-landscapes/guides-manuals-implementation-tools-for-malaria-rdts/ https://www.who.int/malaria/areas/diagnosis/rapid-diagnostic-tests/job-aids/en/

*SOP* Standard Operating Procedure, *Ref material* Reference material

## Conclusions

The WHO strategy is to make RDTs available as close as possible to patients. The tests are often used at community level and results are not confirmed a posteriori, as for tuberculosis or HIV. Total confidence in test results and quality of tests is, therefore, essential. Although tools exist for development of country-level LT, it is unrealistic to expect well-functioning processes to be in place in a near or medium-term in most endemic countries. Regional level capacity may be within reach, but first requires a concerted effort to fund inter-country mechanisms to support it. While the WHO has discussed options for continuation of the programme with key stakeholders, there remains a lack of engagement, heightening the risk of a drop in quality. Continuation of the centralized LT programme would represent a low-cost investment, while in the meantime countries and donors should invest more efforts to develop national or regional capacity. Any form of LT would significantly enhance the overall safeguarding framework, including national regulations and WHO prequalification, to help sustaining the high quality of RDTs.

## Data Availability

Not applicable.

## Abbreviation

EQA: External quality assessment
FIND: Foundation for innovative new diagnostics
GF: The Global Fund to fight AIDS, Tuberculosis and Malaria
GMP: Global malaria programme
HIV: Human immunodeficiency virus
HRP2: Histidine rich protein 2
IPC: Institut Pasteur du Cambodge
IVD: In-vitro diagnostics
LT: Lot testing
RDT: Malaria rapid diagnostic test
MSF: Médecins Sans Frontières
NMCP: National malaria control programme
NRA: National regulatory authority
pLDH: Plasmodium lactate deshydrogenase
PMI: United States President’s Malaria Initiative
PMS: Post-marketing surveillance
PQ: Prequalification of diagnostics
PT: Product testing
RITM: Research institute for tropical medicine
QC: Quality control
QMS: Quality management system
SOP: Standard operating procedures
UNICEF: United Nations International Children’s Emergency Fund
WHO: World Health Organization
